# *Reighardia sternae* Infection and Associated Lesions in a Yellow-Legged Gull (*Larus michahellis*) in Italy

**DOI:** 10.3390/vetsci12050411

**Published:** 2025-04-27

**Authors:** Renato Ceccherelli, Valentina Virginia Ebani, Stefano Pesaro, Giacomo Rossi, Stefania Perrucci

**Affiliations:** 1 Aquatic Bird Recovery Center (CRUMA)-Italian league for bird protection (LIPU) Volunteer organization (ODV), Via Pasubio 3/bis, 43122 Parma, Italy; apusvet.cruma@libero.it; 2 Department of Veterinary Sciences, University of Pisa, 56124 Pisa, Italy; valentina.virginia.ebani@unipi.it; 3 Department of Agricultural, Food, Environmental and Animal Sciences, University of Udine, 33100 Udine, Italy; stefano.pesaro@uniud.it; 4 School of Biosciences and Veterinary Medicine, University of Camerino, 62024 Matelica, Italy; giacomo.rossi@unicam.it

**Keywords:** Italy, pathological findings, Pentastomida, *Reighardia sternae*, yellow-legged gull (*Larus michahellis*)

## Abstract

*Reighardia sternae* is a pentastomid parasitising the respiratory system of larids and skuas, the pathological significance of which is still poorly understood. In this study, pentastomes found in the interclavicular air sac of a deceased and necropsied yellow-legged gull (*Larus michahellis*) were identified by parasitological and molecular methods and associated gross and histopathological lesions were evaluated. After morphological evaluation and molecular characterisation, the species *R. sternae* was identified. This is the first report of *R. sternae* infecting *L. michahellis* in Italy. Gross pathology showed generalised aerosacculitis, parabronchial edema, and enlargement of the heart, liver, and spleen. The systemic lesions found at histopathological examination were indicative of chronic cardiocirculatory failure, which led to the death of the gull. Moreover, inflammation was present throughout the lungs and air sacs in the form of some granulomas and diffuse small nodular aggregates rich in lymphocytes. The remnants of a parasite were observed within a granulomatous lesion in the lung, suggesting a primary host response to the parasite. These latter findings may suggest that the migration of *R. sternae* was the cause of the pathological granulomatous pictures in the examined yellow-legged gull.

## Simple Summary

The pentasomid species *Reighardia sternae* is a blood feeding parasite that can be found in the body cavity, respiratory system, and air sacs of sea birds, the pathological significance of which is still poorly known. An adult male yellow-legged gull (*Larus michahellis*), found injured and later deceased four days after admission to an avian rescue center in central Italy, was submitted for necropsy. Gross examination revealed generalised airsacculitis and cardiac enlargement. In addition, seven morphologically similar female pentastomes were found in the interclavicular air sac and identified as adult females of the species *R. sternae* based on morphological evaluation and molecular characterization. The systemic lesions found in the deceased bird at histopathology were indicative of chronic cardiocirculatory failure that probably caused the death of the gull and could only be secondarily attributed to the presence of the parasite, while a pulmonary granulomatous lesion was indicative of a sequestration by the host of a probable migrating female parasite.

## 1. Introduction

The crustacean subclass Pentastomida comprises over 130 species of obligate parasites, with adult forms typically residing in the respiratory system of vertebrate hosts, including reptiles, amphibians, carnivorous mammals, and birds [1,2,3]. Humans may serve as final or intermediate hosts for some species [4,5]. Most pentastomid species have an indirect life cycle involving intermediate and final hosts and are found in reptiles [3,6]. Only four pentastomid species have been reported to parasitise the respiratory system of birds [2,7]: *Reighardia sternae* in gulls, terns and other larids, as well as in skuas [2,7]; *Reighardia lomviae* in some Charadriiformes species of the family Alcidae (*Uria aalge*, *Uria lomvia Fratercula arctica*, *Fratercula cirrhata Synthlibor amphus antiquus*, *Aethia cristatella*) [2,7]; *Hispania vulturis* in the black vulture (*Aegypius monachus*) [8]; *Raillietiella trachea* in the white-rumped vulture (*Gyps bengalensis*) [9].

The globally distributed species *R. sternae* is a blood-feeding parasite that can be found in the body cavity, respiratory system, and air sacs of seabird hosts [10]. The life cycle of *R. sternae* does not involve intermediate hosts as transmission is direct, mainly following the ingestion of larvated eggs [10,11,12,13]. In newly infected avian hosts, digestive processes lead to the rupture of *R. sternae* eggshells, allowing the larvae to hatch, penetrate the intestinal wall, and migrate to the body cavity. There they feed on ruptured intestinal capillaries. Sexual differentiation and mating take place within a few weeks, and spermatozoa are stored in two seminal receptacles of the females. After mating, males can be found in the air sacs flanking the viscera, including the posterior thoracic and abdominal air sacs. They can also be found in the body cavity—on the mesenteries, intestines, and occasionally around the kidneys—where they eventually die [11,14,15]. Females enter the abdominal air sacs and begin their migration moving via the thoracic air sacs, or via the bronchioles, to the interclavicular air sac, feeding on the capillaries of the air sac layer. The eggs develop synchronously. After a pre-patent period of about six months, the females undergo a second migration from the interclavicular air sacs to the buccal cavity to lay eggs [11,15]. The eggs and whole or part of the female are regurgitated or swallowed and excreted by the bird host, becoming a source of infection for other birds [11,15]. Indeed, it is thought that migrating patent females induce vomiting in gulls, and transmission in larids occurs mainly through the ingestion of egg-contaminated vomit by other gulls or during chick feeding [2,12]. In skuas, predation of infected gulls and terns may also serve as a potential route of transmission [16].

*Reighardia sternae* generally shows a low prevalence, and although auto-reinfection has been reported, repeated infections are typically limited by the development of an immune response [12,15,17].

Although *R. sternae* has not previously been considered a parasite of pathological significance [18], *R. sternae* infection associated with air sacculitis and lung lesions has recently been reported in a free-ranging Herring gull (*Laurus argentatus*), suggesting that this parasite may have significant detrimental effects in infected marine birds [19].

The present study reports the first record of *R. sternae* in the air sacs of a deceased yellow-legged gull (*Larus michahellis*) in Italy and the associated lesions found in the deceased bird.

## 2. Materials and Methods

### 2.1. Case Description and Gross Lesions

**Figure 1:**
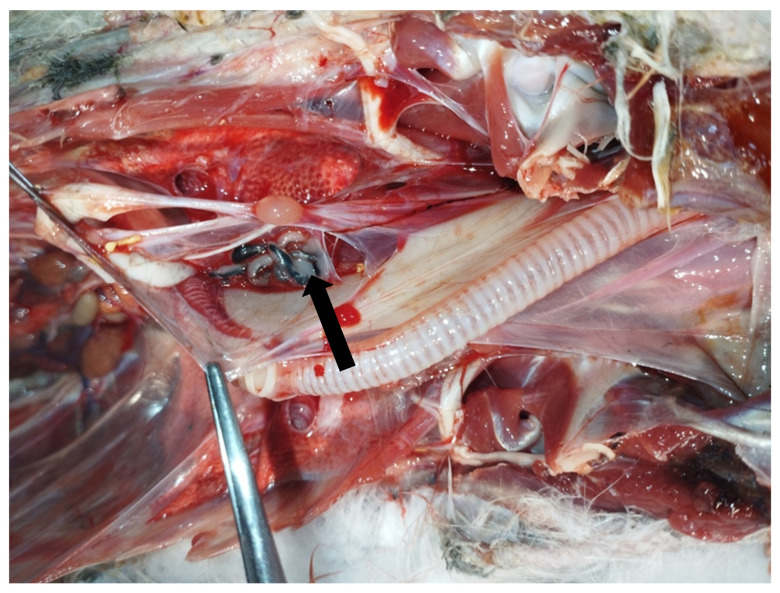
Necropsy of a deceased yellow-legged gull (*Larus michahellis*) showing the presence of pentastomes (arrow) in the interclavicular air sac.

**Figure 2:**
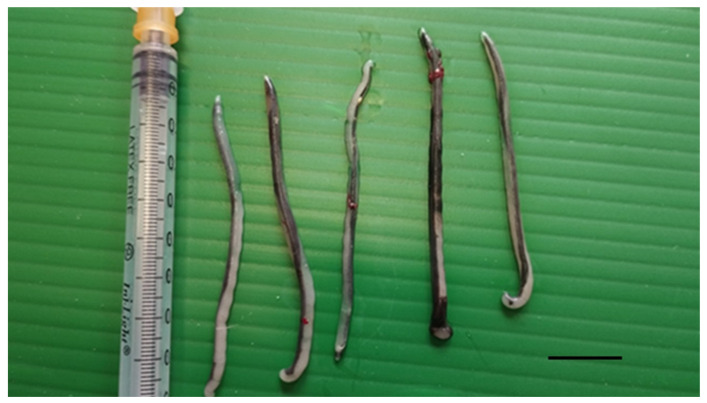
Parasites collected from the interclavicular air sac of a deceased yellow-legged gull. Scale bar 1 cm.

In May 2023, an adult male yellow-legged gull (*Larus michahellis*) found in poor general condition in the city of Livorno, Tuscany, Central Italy, was hospitalized at the Marine and Aquatic Bird Recovery Center (CRUMA) managed by the Italian league for bird protection (LIPU) of Livorno. On clinical examination, the bird was found to be hyporeactive, dehydrated, and unable to eat or drink water, exhibiting clinical signs consistent with a paretic syndrome. Body temperature was 37.9 °C. Intravenous fluid therapy and parenteral nutrition were started, and the bird was placed under a hot lamp. Despite an initial improvement and an increase in body temperature to 39.2 °C, the bird deteriorated rapidly and died four days after admission. Necropsy revealed generalised air sacculitis, parabronchial oedema, and enlargement of the heart, liver, and spleen. Moreover, seven morphologically similar pentastomes were found in the interclavicular air sac at the level of the sub-cardiac diverticulum (Figure 1 and Figure 2).

### 2.2. Morphological Identification of Parasites

The seven collected pentastome specimens were rinsed in physiological saline to remove debris. Their total length and width were measured, after which they were preserved in 70% ethanol for further analysis. Two were used for molecular analysis. For microscopic analysis, the remaining five specimens were cleared using lactophenol and examined. Morphological and metrical analysis of the whole parasites, parasite internal organs, eggs, cephalic hooks, and oral apparatus were specifically performed. Measurements were taken with an ocular micrometer. Morphological identification was performed according to the description given by Kanarek et al. [20], Naupay et al. [3] and Literak et al. [2].

### 2.3. Molecular Analysis

Two portions of the posterior part of the parasite body were submitted to DNA extraction. The commercial kit DNeasy Tissue kit (Qiagen GmbH, Hilden, Germany) was used following the manufacturer’s instructions. Extracted DNA was stored at 4 °C until used as template for the PCR assay.

PCR was carried out using the primers, targeting a 383 bp fragment of the nuclear 18S rDNA locus, Pent629F (CGGTTAAAAAGCTCGTAGTTGG), and Pent101IR (GGCATCGTTTATGGTTAGAACTAGGG) [21].

PCR reactions were assessed in a final volume of 25 µL, containing 12.5 µL of EconoTaq PLUS 2X Master Mix (Lucigen Corporation, Middleton, WI, USA), 0.1 µM of each primer, 2 µL of extracted DNA, and ultrapure water to reach the final volume. Sterile distilled water was added as negative control.

PCR amplification was performed in a thermal cycler (PCR Sprint, Thermo Hybaid, Franklin, MA, USA) using PCR conditions previously reported by Brookins et al. [21]. In detail, the protocol included 5 min at 95 °C for the initial denaturation, followed by 40 cycles of 95 °C for 1 min, 58 °C for 1 min, and 72 °C for 1 min; a final step of 10 min at 72 °C completed the reaction.

The PCR products were analysed by electrophoresis on a 1% agarose gel and stained with ethidium bromide at 100 V for 45 min. SharpMass™ 100 Plus Ladder (Euroclone, Milano, Italy) was used as DNA marker.

The 383 bp band of each extracted sample was excised and purified using the QIAquick gel extraction kit (Qiagen) and the obtained products were sequenced by a commercial laboratory (BMR-Genomics, Padova, Italy). The sequences were assembled and corrected by visual analysis of the electropherogram using Bioedit v.7.2, and then compared with those available in GenBank using the BLAST program 2.15.0 (http://www.ncbi.nlm.nih.gov/BLAST, accessed on 10 January 2024).

### 2.4. Histopathological Analysis

Liver, spleen, kidneys, heart, lungs, air sacs, gastrointestinal tract, bursa of Fabrizio, large vessels, reproductive system, and central nervous system tissues from the necropsied yellow-legged gull were fixed in 10% neutral buffered formalin for at least 2 days before trimming into cassettes, embedding in paraffin wax, sectioning by microtome into 3-µm ribbons, mounting onto glass slides, and staining with hematoxylin and eosin for standard light microscopy observation. Ziehl–Neelsen, PAS, and Grocott’s stains were also performed. Spleen sections were also stained with Congo red and examined by polarised light microscopy.

## 3. Results

### 3.1. Morphological Identification of Parasites

On morphological analysis, the parasites found in the air sacs of the deceased *L. michahellis* examined in this study were identified as mature female pentastomids of the species *R. sternae*.

**Figure 3:**
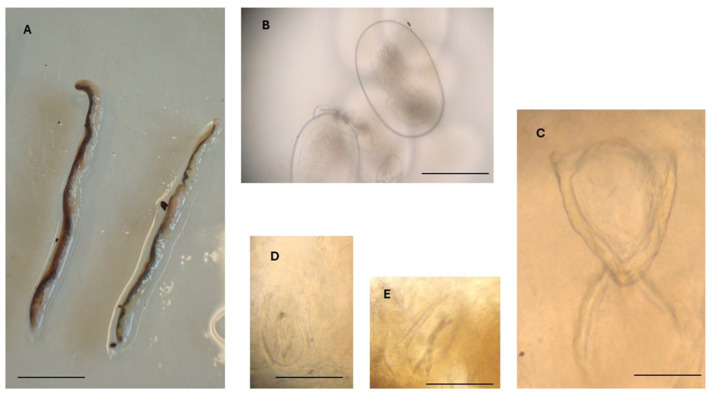
*Reighardia sternae* found in the interclavicular air sacs of a yellow-legged gull (*Larus michahellis*). (**A**) Adult female specimens, scale bar = 1.0 cm. (**B**) Egg containing a larva, scale bar = 150 μm. (**C**) Oral apparatus, scale bar = 150 μm. (**D**) Right anterior hook, scale bar = 50 μm. (**E**) Left posterior hook, scale bar = 50 μm.

Parasite bodies (n = 7) were elongated and slender, with a total body length ranging from 44 to 49 mm (mean 46.6 mm) and a maximum width range of 1.9–2.7 mm (mean 2.4) (Table 1; Figure 3A).

In all specimens metrically examined (n = 5), the genital organs contained numerous larvated eggs measuring 260–320 µm (mean 281 µm) in length and 150–190 µm in width (mean 170 µm) (Table 1; Figure 3B).

The oral apparatus (Figure 3C) was in the anterior region of the body and consisted of an oral opening, a V-shaped oral cadre with sclerotized structures, a pharynx, and two posterior crescentic structures. The length of the oral apparatus was about 285 µm (Table 1).

Posterior to the oral apparatus, two pairs of hooks were visible, one pair anterior to the other pair. The two hooks of each pair were located one on the left and the other on the right side of the anterior portion of the body. Anterior hooks were 90–110 µm long (mean 97 µm), while posterior hooks were 95–110 µm long (mean 105 µm) (Figure 3D,E).

According to morphological features, the pentastomes were identified as adult females of *R. sternae*, and all morphometric characteristics were in good agreement with those reported for *R. sternae* by Kanarek et al. [20], Naupay et al. [3], and Literák et al. [2] (Table 1).

### 3.2. Molecular Identification

**Figure 4:**
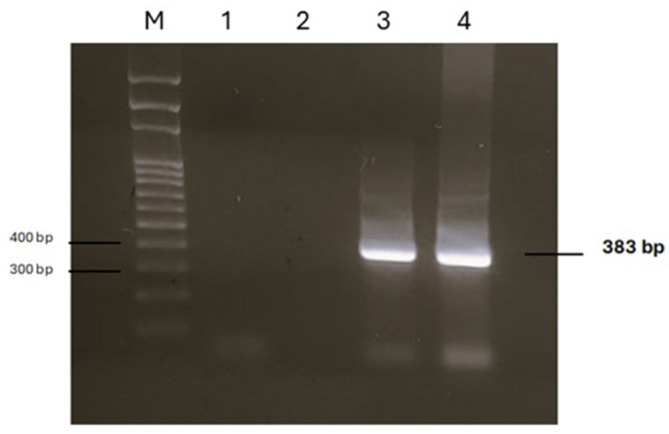
Agarose gel of PCR products obtained by analysing DNA extracted from two parasites. M: marker SharpMass™ 100 Plus Ladder (Euroclone, Milan, Italy); lanes 1 and 2: negative controls; lanes 3 and 4: positive samples.

**Figure 5:**
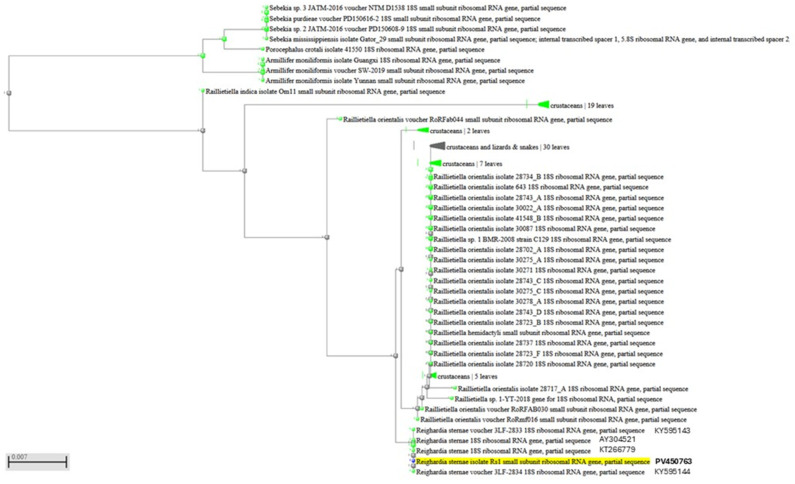
Dendrogram displaying genetic relationships of the PV450763 sequence analyzed in this study with other *Reighardia sternae*.

DNA samples extracted from two portions of two parasites allowed the amplification of a 383 bp product. Sequencing analysis of both amplicons identified the parasite as *R. sternae*, with a 100% sequence homology to *R. sternae* previously found in *Larus ridibundus* in Spain (accession number AY304521), *Larus michahellis* and *Larus fuscus* in Portugal (accession numbers KY595143 and KY595144), and *Larus belcheri* in Peru (accession number KT266779) [2,3]. A single sequence was deposited in the GenBank database under the accession number PV450763. Figure 4 shows the PCR products, whereas the dendrogram displaying the genetic relationships with other *R. sternae* is reported in Figure 5.

### 3.3. Histopathological Findings

**Figure 6:**
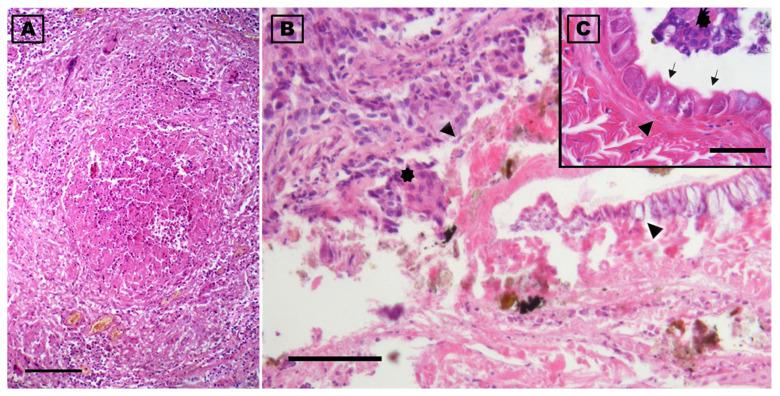
Photomicrographs of the lung of a deceased yellow-legged gull (*Larus michahellis*) in central Italy found infected with *Reighardia sternae*. (**A**) Cross section of the granulomatous lesion present in the lung. (**B**) Note at the highest magnification the presence of inflammatory exudate which, in the center of the granuloma, is organized around the fragments of the parasite (arrowheads). (**C**) Detail of a cuticle fragment showing the striations, glands (arrowhead), and some tubercle parts (arrows) are evident; multinucleated giant cells of the inflammatory reaction are noted in (**B**) and (**C**) (asterisks). Hematoxylin-eosin stains. Scale-bars: 500 µm (**A**), 100 µm (**B**), and 50 µm (**C**).

Histologically, pulmonary oedema was present, in the form of serum-proteinaceous material at the level of the parabronchi. Additionally, protein-rich fluids and small numbers of inflammatory cells were present in the airways of the yellow-legged gull. Both at the interstitial level in the lung parenchyma and free in the airways groups of macrophages containing brown or golden yellow granular hemosiderin pigment were occasionally seen, indicating repeated micro-hemorrhagic phenomena preceding death. At the level of the respiratory epithelium, few granulocytes, macrophages, and lymphocytes were present among the alveolocytes or in the lamina propria. Inflammation was present throughout the lung and air sacs, in the form of small nodular aggregates rich in lymphocytes in the bronchioles and parabronchi. Ziehl–Neelsen, PAS, and Grocott’s stains did not reveal the presence of alcohol–acid-fast bacteria or fungal hyphae. In one area of the lung there was a granulomatous lesion with a core of necrotic debris bordered by multinucleated giant cells and macrophages and surrounded by many lymphocytes; the material in the core of the granuloma was pale pink, hyaline, finely stratified, and partially mineralised, with the presence of some fragments of a cuticular structure. This cuticle preserved fragments of muscle and some integumentary tubercles and could be associated with parts of a migrating parasite (Figure 6).

The myocardial wall of the left ventricle appeared hypertrophic, with some groups of fibers with a necrotic appearance (Zenker’s necrosis). The spleen also appeared greatly enlarged in volume, characterised by hyperplasia of the white pulp and by the deposition of an amorphous, weakly eosinophilic and congophilic substance of amyloid nature, associated with a chronic stimulation of the immune system and related to excessive production of immunoglobulins. Finally, a widespread liver pathology, characterised by diffuse fibrosis and fatty dystrophy of the hepatocytes, was attributed to a generalized stasis linked to progressive cardiovascular failure. Fibrosis and diffuse deposits of sodium urate were also observed in numerous renal tubules. All other examined organs were normal in appearance. The lesions observed in the spleen, liver, kidneys, and heart are typical systemic lesions related to chronic cardiocirculatory failure, with increased stasis at the level of the various parenchymas, fibrosis, and further increase in peripheral circulatory resistance. Therefore, cardiocirculatory failure was considered to be the main cause of death of the gull.

## 4. Discussion

*Reighardia sternae*, also known as the larid pentastome, is the only pentastomid species to use gulls and terns as hosts, living in the body cavity and air sacs [2]. This parasite has been reported in more than 20 species of birds of the genera *Chlidonas*, *Chroicocephalus*, *Fratercula*, *Larus*, *Leucophaeus*, *Pagophila*, *Rissa*, *Sterna*, and *Uria* [2,3,7]. Most of *R. sternae* infections have been recorded in different geographical areas in the Northern Hemisphere, including Europe, Japan, and the United States of America [1,2,11,14,20,23,24,25]. Few reports are available about the occurrence of *R. sternae* in the Southern Hemisphere where this pentastome species has been reported in the Dominican gull (*Larus dominicanus*), in the Belcher’s gulls (*Larus belcheri*), and in the South Polar skua (*Catharacta maccormicki*) [3,26,27].

According to the life cycle of the parasite [11,14,15], only mature females can be found in the interclavicular air sac, as evidenced in this study and in previous studies [14,20]. Variations in the body length of adult females of *R. sternae* have been reported in previous investigations, ranging from 30 to 46 mm according to Riley [22], 43.7 to 53.9 mm according to Karanek et al. [20], 33.3–34.8 mm according to Naupay et al. [3], and 51–54 mm according to Literák et al. [2]. However, these variations could be related to the stage of development of adult females in the infected bird host or to intraspecific variation or, again, to the fixation method used on such specimens [2,20]. Nevertheless, the dimensions of *R. sternae* adult females found in this study (length 44 to 49 mm) and other morphological features totally overlap those reported by Riley [22] and Karanek et al. [20] and are in line with those reported by Naupay et al. [3] and Literák et al. [2].

Moreover, sequencing analyses revealed a 100% sequence homology of pentastomes here reported to *R. sternae* previously found in *L. ridibundus* in Spain, *L. michahellis* and *L. fuscus* in Portugal, and to *Reighardia* sp. found in *L. belcheri* in Peru [2,3].

In *L. michahellis*, *R. sternae* was recently reported only in Portugal [2]. Here, we report for the first time *R. sternae* infecting *L. michahellis* in Italy.

The yellow-legged gull is a typical species of the Mediterranean area, including Italy, where the bird nests widely along the coast, but its populations are also present on the Atlantic coasts of France and Spain, as well as in the Black Sea and North Africa [28]. The Italian population is mainly sedentary, although in the post-breeding season some birds can move some distances. In the city of Livorno, an extensive colonization of the urban area has been highlighted [29]. Therefore, it seems that the gull could have been infected in Italy.

The prevalence of *R. sternae* infection is generally low, in gulls of the genus *Larus*, ranging from 0.5–8.9% in *L. ridibundus* [30,31,32,33], 5.2–7.9% in *L. canus* [15,25], 4.8–10% in *L. argentatus* [14,20,32], 8–12.3% in *L. marinus* [15,32], and 3% in *L. fuscus* [2], 4% in *L. michahellis* [2]. Prevalence is higher in juveniles than in adults, suggesting an age-dependent immunity [15,20,25]. Furthermore, massive infections are relatively uncommon, and parasite burden is often less than 10 parasites per infected bird [20], as shown in this study in which seven parasites were found in the infected yellow-legged gull. Young, immature birds are generally more heavily infected than adults [15,20,25].

Although most pentastome species may cause pathological damage, often associated with parasite migration and with the hooks and mouths of feeding adults [34], *R. sternae* is not regarded as a parasite of pathological significance or a primary cause of mortality in marine birds [18]. However, *R. sternae* was recently reported in association with severe granulomatous air sacculitis and extensive lung lesions in a free-ranging herring gull (*L. argentatus*), suggesting that this parasite may have significant detrimental effects in infected birds [19].

In the presented case, a generalized air sacculitis was demonstrated. Nevertheless, the systemic lesions found in *L. michahellis* were indicative of chronic cardiocirculatory failure that probably caused the death of the bird. In fact, the lesions at the liver and kidney level are typically related to a primary blood circulation disorder and hypertension/stasis, as indicated by pulmonary erythrophagocytosis and the presence of siderocytes [35]. These lesions were associated with chronic inflammatory granulomatous and fibrotic alterations at the pulmonary level, which may have induced an increase in cardiac work with final failure. Moreover, in the absence of aspergillosis and specific bacterial infections, such as mycobacteria, the observation of diffused lung and air sac inflammation in the form of small nodular aggregates rich in lymphocytes is not a common finding in seagulls and we presume that it may be a consequence of the parasitic presence, since this kind of lesion has been reported for pentastomes in other animal species [36]. Despite the involvement of other pathogens as concurrent agents cannot be completely excluded, this widespread inflammation at the level of the lung parenchyma and air sacs was probably caused by the migration of *R. sternae* and could have contributed to the alteration of the blood pressure level of the small circulation and to cardiac hypertrophy and cardiovascular failure. Moreover, the lung granulomatous lesion, indicating host sequestration of a probable migrating female parasite, could be related to a primary host response to the parasite.

## 5. Conclusions

In conclusion, morphological and molecular analysis allowed the identification of the pentastomid species *R. sternae*. The present report is the first record of *R. sternae* in a deceased yellow-legged gull (*L. michahellis*) in Italy. Further epidemiological studies are needed to determine the diffusion of this pentastome species across the Italian territory. Data from this study may suggest that the pneumo-oral migration of *R. sternae* was a cause of pathological granulomatous pictures in the examined *L. michahellis*. Nevertheless, further investigations are needed to evaluate the pathological significance of this pentastomid species for the seabird hosts.

## Figures and Tables

**Table 1:** Morphometric data of *Reighardia sternae* females found in this study and according to various authors.

Parasite	*Reighardia sternae* *Larus michahellis* This Study	*Reighardia sternae* *Larus michahellis* and *Larus fuscus* Literák et al. [2]	*Reighardia sternae* *Larus argentatus* Kanarek et al. [20]	*Reighardia* sp. *Larus belcheri* Naupay et al. [3]	*Reighardia sternae* *Larus argentatus* Riley [22]
Body length (mm)	44–49 (mean 46.6)	51–54 (mean 52)	43.7–53.9	33.3–34.8 (mean 33.9)	30–46
Body width (mm)	1.9–2.7 (mean 2.4)	2.8–3.1 (mean 2.9)	1.89–2.53	1.9–2.2 (mean 2)	2.5
Eggs (µm) (Length and width)	260–320 (mean 281) in length and 150–190 in width (mean 170)	316.5–381.7 (mean 353.9) in length and 233.6–269.9 (mean 250.4)	260–380 in length 140–290 in width	-	-
Oral apparatus (µm) (Length)	285	295.4–303.4 (mean 298.7)	-	-	-
Anterior hooks (µm) (Length)	90–110 (mean 97)	94.6–109.5 (mean 102)	111–125	97–105 (mean 102)	87–100
Posterior hooks (µm) (Length)	95–110 (mean 105)	100.5–109.1 (mean 104.3)	87–108	100–105 (mean 103)	92–106

## Data Availability

The raw data supporting the conclusions of this article will be made available by the authors on request.

## References

[1] Poore G.C. (2012). The nomenclature of the recent Pentastomida (Crustacea), with a list of species and available names. Syst. Parasitol..

[2] Literák I., Casero M., Koubková B., Těšínský M., Heneberg P. (2017). Morphological and Molecular Assessment of Pentastomes from Gulls in Portugal. J. Parasitol..

[3] Naupay A.I., Cribillero N.G., Lopez-Urbina M.T., Gonzalez A.E., Gomez-Puerta L.A. (2016). Finding of pentastomes of genus *Reighardia* (Pentastomida) in the Belcher’s gull (*Larus belcheri*). Parasitol. Int..

[4] Hajipour N., Ghorani M., Ketzis J. (2025). Phylogenetic, molecular, and microscopic investigation of *Linguatula serrata* infection in stray and road-killed dogs in Northwest Iran. BMC Vet. Res..

[5] Barton D.P., Shamsi S. (2024). Diagnosis of Pentastome Infections and the Need for Increased Awareness Among Medical Practitioners and Diagnosticians in the Developed World. Curr. Clin. Microbiol. Rep..

[6] Riley J. (1986). The biology of pentastomids. Adv. Parasitol..

[7] Christoffersen M.L., De Assis J.E. (2013). A systematic monograph of the recent Pentastomida, with a compilation of their hosts. Zool. Mededel..

[8] Martínez J., Criado-Fornelio A., Lanzarot P., Fernández-García M., Rodríguez-Caabeiro F., Merino S. (2004). A new pentastomid from the black vulture. J. Parasitol..

[9] Riley J., Oaks J.L., Gilbert M. (2003). *Raillietiella trachea* n. sp., a pentastomid from the trachea of an oriental white-backed vulture *Gyps bengalensis* taken in Pakistan, with speculation about its life-cycle. Syst. Parasitol..

[10] Riley J. (1973). The structure of the buccal cavity and pharynx in relation to the method of feeding of *Reighardia sternae* Diesing 1864 (Pentastomida). Int. J. Parasitol..

[11] Banaja A.A., James J.L., Riley J. (1975). An experimental investigation of a direct lifecycle in *Reighardia sternae* (Diesing, 1864), a pentastomid parasite of the herring gull (*Larus argentatus*). Parasitology.

[12] Banaja A.A., James J.L., Riley J. (1976). Some observations on egg production and autoreinfection of *Reighardia sternae* (Diesing, 1864), a pentastomid parasite of the herring gull. Parasitology.

[13] Wernery U., Zwart P., Schuster R., Krone O., Peirce M.A., Coutteel P., Wencel P., Samour J. (2016). Chapter 14—Infectious Diseases. Avian Medicine.

[14] Riley J. (1972). Some observations on the life-cycle of *Reighardia sternae* diesing 1864 (pentastomida). Z. Parasitenk..

[15] Bockeler W. (1984). Der Entwicklungszyklus von *Reighardia sternae* (Pentastomida) nach Untersuchungen an natürlich und experimental infestierten Möwen. Zool. Anz..

[16] Vanstreels R.E.T., Palma R.L., Mironov S.V. (2020). Arthropod parasites of Antarctic and Subantarctic birds and pinnipeds: A review of host-parasite associations. Int. J. Parasitol. Parasites Wildl..

[17] Riley J., James J.L., Banaja A.A. (1979). The possible role of the frontal and sub-parietal gland systems of the pentastomid *Reighardia sternae* (Diesing, 1864) in the evasion of the host immune response. Parasitology.

[18] Brosens L., Jauniaux T., Siebert U., Benke H., Coignoul F. (1996). Observations on the helminths of harbour porpoises (*Phocoena phocoena*) and common guillemots (*Uria aalge*) from the Belgian and German coasts. Vet Rec..

[19] Jansson D.S., Bröjer C., Neimanis A., Mörner T., Murphy C.L., Otman F., Westermark P. (2018). Post mortem findings and their relation to AA amyloidosis in free-ranging Herring gulls (*Larus argentatus*). PLoS ONE.

[20] Kanarek G., Rolbiecki L., Misztal M. (2005). *Reighardia sternae* (Diesing, 1864) a pentastomid (Pentastomida) species new for the fauna of Poland. Fragm. Faun..

[21] Brookins M.D., Wellehan J.F., Roberts J.F., Allison K., Curran S.S., Childress A.L., Greiner E.C. (2009). Massive visceral pentastomiasis caused by *Porocephalus crotali* in a dog. Vet Pathol..

[22] Riley J. (1973). A redescription of *Reighardia sternae* Diesing 1864 (Pentastomida: Cephalobaenida) with some observations on the glandular systems of pentastomids. Z. Für Morphol. Tiere.

[23] Ooi H.K., Ohbayashi M. (1982). *Reighardia sternae*, a pentastomid froma slaty-backed gull in Hokkaido, Japan. Jpn. J. Vet. Res..

[24] Pence D.B. (1973). *Reighardia sternae* (Cephalobaenida: Reighardiidae), a pentastome from gulls and terns in Lousiana. Proc. Helminthol. Soc. Wash..

[25] Bakke T.A. (1972). *Reighardia sternae* (Diesing, 1864), Ward, 1899 (Pentastomida: Cephalobaenida) from the common gull (*Larus canus* L.) in a Norwegian locality. Norw. J. Zool..

[26] Hoberg E.P. (1987). *Reighardia sternae* (Diesing, 1864) (Pentastomida) from seabirds in Antarctica. Can. J. Zool..

[27] Rego A.A. (1984). Sinopse dos pentastomídeos da região neotropical. Garcia Orta Ser. Zool..

[28] Alonso H., Almeida A., Granadeiro J.P., Catry P. (2015). Temporal and age-related dietary variations in a large population of yellow-legged gulls *Larus michahellis*: Implications for management and conservation. Eur. J. Wildl. Res..

[29] Franceschi A. (2013). Distribution and consistency of Yellow-legged Gull, *Larus michahellis*, along the coastal shore in continental Tuscany. Riv. Ital. Ornitol..

[30] Pemberton R.T. (1963). Helminth parasites of three species of British gulls, *Larus argentatus* Pont., *L. fuscus* L. and *L. ridibundus* L.. J. Helminthol..

[31] Creutz G.V., Gottshalck C. (1969). Endoparasitenbefall bei Lachmöwen in Abhängigkeit vom Alter. Angew. Parasitol..

[32] Böckeler W., Vauk-Hentzelt E. (1979). Die Mantelmöve (*Larus marinus*) als neuer Wirt des Luftsackpiraten *Reighardia sternae* (Pentastomida). Zool. Anz..

[33] Barus V., Sitko J., Tenora F. (1978). Nematoda and Pentastomida parasitizing gulls (Aves: Laridae) in Bohemia and Slovakia. Acta Univ. Agric. Fac. Agron..

[34] Kelehear C., Spratt D.M., O’Meally D., Shine R. (2013). Pentastomids of wild snakes in the Australian tropics. Int. J. Parasitol. Parasites Wildl..

[35] Ruffenach G., Hong J., Vaillancourt M., Medzikovic L., Eghbali M. (2020). Pulmonary hypertension secondary to pulmonary fibrosis: Clinical data, histopathology and molecular insights. Respir. Res..

[36] Ambrose N.C., Riley J. (1988). Light microscope observations of granulomatous reactions against developing *Porocephalus crotali* (Pentastomida: Porocephalida) in mouse and rat. Parasitology.

